# Prelude to a Compositional Systems Biology

**Published:** 2024-08-01

**Authors:** Eran Agmon

**Affiliations:** 1 Center for Cell Analysis and Modeling, Department of Molecular Biology and Biophysics, University of Connecticut Health, Farmington, Connecticut, USA.

**Keywords:** systems biology, multiscale modeling, simulation, interfaces, composition

## Abstract

Composition is a powerful principle for systems biology, focused on the interfaces, interconnections, and orchestration of distributed processes. Whereas most systems biology models focus on the structure or dynamics of specific subsystems in controlled conditions, compositional systems biology aims to connect such models into integrative multiscale simulations. This emphasizes the *space between models*—a compositional perspective asks what variables should be exposed through a submodel’s interface? How do coupled models connect and translate across scales? How can we connect domain-specific models across biological and physical research areas to drive the synthesis of new knowledge? What is required of software that integrates diverse datasets and submodels into unified multiscale simulations? How can the resulting integrative models be accessed, flexibly recombined into new forms, and iteratively refined by a community of researchers?

This essay offers a high-level overview of the key components for compositional systems biology, including: 1) a conceptual framework and corresponding graphical framework to represent interfaces, composition patterns, and orchestration patterns; 2) standardized composition schemas that offer consistent formats for composable data types and models, fostering robust infrastructure for a registry of simulation modules that can be flexibly assembled; 3) a foundational set of biological templates—schemas for cellular and molecular interfaces, which can be filled with detailed submodels and datasets, and are designed to integrate knowledge that sheds light on the molecular emergence of cells; and 4) scientific collaboration facilitated by user-friendly interfaces for connecting researchers with datasets and models, and which allows a community of researchers to effectively build integrative multiscale models of cellular systems.

## 1 Introduction

Cellular systems are multimodal and multiscale, with diverse mechanisms operating across many levels of organization. Their collective behavior, and connection to an environment, drives the evolution of dynamic self-organizing hierarchies—the biosphere being made of a bewildering number of cells that adapt, grow, and evolve within their environments, which drives the emergence of structures such as communities, tissues, organisms, ecosystems—and each cell itself a multilayered structure made of molecules, complexes, condensates, organelles. Modeling such systems demands a shift from examining individual subsystems in isolation to an integrative approach that emphasizes the interfaces of these subsystems and how they connect. The complexity of cells, together with the large number of researchers needed to thoroughly analyze even a single cellular subsystem, underscores the need for an open, collaborative scientific framework in which new data and models can be combined and recombined in the synthesis of new biological knowledge.

Compositionality is offered as an overarching principle to facilitate the integration of diverse datasets and models into open-ended simulations of cellular systems. This use of compositionality is adapted from category theory [1] and software design [2], but in this essay reinvisioned for systems biology. Three fundamental criteria underpin compositionality. First, the **interfaces of subsystems**, which act as points of interaction. Second, a **composition pattern** linking different subsystems through their interfaces. Third, an **orchestration pattern** that drives composition in time by coordinating the subsystems’ activities, which include modifications to the composition pattern itself. For a complete definition of a composite system, each of these criteria requires a clear, standardized specification that enables model reproduction, sharing, iteration, and extension. These should come bundled as a set of interoperable process interfaces, composition patterns, and orchestration patterns—this set of three will here be referred to as a **composition protocol**.

Composition pervades biology. At the molecular scale, cells are composed of a vast number of molecules that interact through their specific interfaces to drive changes to their structures, functions, and surroundings. The particular molecular composition of a cell determines its interface with the environment, through which it exchanges the material, energy, and information required to sustain itself and drive its growth. These interactions are constantly changing, as cells respond to signals, compete for resources, cooperate with other cells, and restructure their very environment. At higher levels of organization, populations of cells compose into communities, organisms, up to ecosystems. The patterns of how cellular interfaces connect with each other and with their environments determines their chemical, electrical, and mechanical inputs, driving their behavior, and setting the course of their evolution.

Systems biology models have not been compositional in the same way as their biological counterparts. This is in large part because they do not have chemistry and physics to mediate their interactions, and require a model for every natural process. It is true that some modeling paradigms such as ordinary differential equations (ODEs), flux balance analysis (FBA), Bayesian networks, rule-based models, and Markov models are individually compositional in that they can be expanded by adding new expressions or nodes. But different aspects of biology are typically studied with different modeling paradigms, and these too need to be brought together. The need to connect models across scales has driven interest in hybrid approaches that combine modeling paradigms—such as stochastic with deterministic [3], kinetic ODEs with steady state FBA [4, 5], particle-based with continuous spatial [6], and whole-cell models with many interacting processes [7, 8] including connecting kinetic model outputs to 3D spatial models with molecular resolution [9, 10, 11]. Multicellular models often use agent-based models (ABMs) [12, 13, 14]—a class of hybrid models that span two scales, with a model of an environment and a model of individual agents. However, most hybrid models are usually built ad-hoc or hard-coded in their own closed software ecosystem, without an easy way to flexibly expand and connect with external models that use different methods.

To address the need to easily integrate diverse models and methods into cohesive, multiscale simulations, we the developed the Vivarium software [15]. Vivarium has been applied to a range of domains, including the integration of FBA with kinetic ODEs and agent-based modeling [15], bacterial chemotaxis that combines motile forces with a chemical reaction network [16], agent-based modeling of tumor morphogenesis integrated with multiplexed spatial imaging [17], biophysical models of actin at multiple scales [18], and a comprehensive whole-cell model of *E. coli* that combines FBA, ODEs, and stochastic algorithms [19]. Building on the work of earlier frameworks such as ProMoT/Diva [20], MUSCLE 3 [21, 22], Ptolemy II [23], and Modelica’s Functional Mock-up Interface [24], Vivarium revitalizes these efforts by introducing a hierarchical structure and process-centric design that not only unifies multi-scale [25] and middle-out [26] approaches but also facilitate the integration of previously isolated simulation tools [27]. Figure 1 illustrates a conceptual design of the user interface for Vivarium, showcasing how cellular systems can be modeled through the composition of submodels connected by wires, and provide intuitive integration across scales.

Vivarium’s composition framework, called process bigraphs, is being extended with the process-bigraph software suite—to be introduced in a separate more technical paper, so that this essay can focus more on composition in a more general manner. This essay outlines the core requirements of a successful compositional systems biology and proposes its initial instances with templates for molecular and cellular compositions. This sets a foundation for robust simulation infrastructure, and also provide useful conceptual tools for thinking about cellular systems. These topics are described in the following sections:

- **Section 2: Composition framework.** Introduces the basic concepts of process bigraphs, how interfaces work, and how they connect in nested composite systems. Rather than going into the framework’s formal underpinnings, diagrams are used to graphically represent the concepts—these provide a visual language that will be reused to build more complex concepts throughout the essay. Central to a composition framework is a standardized protocol that governs how data, models, and software communicate, setting the foundation for infrastructure that can support an open-ended modeling ecosystem.
- **Section 3: Templates for multiscale cellular modeling.** Describes the cellular interface and analyzes how this interface can emerge through the composition of molecular mechanisms. This provides a compositional perspective on the emergence of cells, how they navigate viability, how they grow, divide, develop into multilayered dynamic organizations, how they ultimately disintegrate back into molecules, and how this ongoing composition drives evolution. These schemas can be thought of as templates for a longer-term goal, to fill in detailed mechanisms that through co-simulation will recapitulate the behavior of real biological cells.
- **Section 4: Collaborative biosciences.** Discusses the impact compositional thinking can have on systems biology as a field, focusing on knowledge integration through intuitive human interfaces and collaborative practices. By embracing compositionality in our research communities, we can enhance data and model integration, as well as pool expertise for a unified biological understanding.

By emphasizing the composition of data, models, schemas, software, and research efforts, we are taking important steps towards a more compositional systems biology. Such a framework not only facilitates the integration of diverse biological data and models but also ensures that these drive the synthesis of new knowledge, and can evolve with new scientific insights.

## 2 Composition framework

To ensure the ongoing scalability of systems biology models, we need to design a robust and general-purpose framework that can scale to any biological problem. This would need to be intuitive to implement, easy to use, and compatible with any modeling effort across systems biology—supporting the integration of any biological dataset and model. Key features of a composition framework include standardized **process interfaces**, **composition patterns**, and **orchestration patterns**. This section offers a high-level overview of these requirements, setting aside a detailed description of the formal system for another paper, and instead relying on **composition diagrams** to visually represent the different components of a compositional model, including processes, ports, states, connections, and nesting.

This essay focuses on a composition framework called “process bigraphs”, which is an evolutionary extension of Robin Milner’s **bigraphs** [28] (Fig. 2A), and was initially implemented by Vivarium [15]. Bigraphs are a powerful framework for compositional modeling due to their ability to represent complex systems through hierarchical structures and flexible reconfigurations, related to agent-based modeling. A bigraph combines two distinct graph structures: a **place graph** (shown in Fig. 4B), which represents the hierarchical nesting of entities within other entities, and a **link graph**, which represents the connectivity of a system using hyperedges to capture the communication between multiple entities. **Process bigraphs** (Fig. 2B) reimagine this structure with the introduction of processes, replacing the link graph with a process-state interaction graph, more simply called a **process graph** (shown in Fig. 5A). Processes introduce additional considerations for orchestration—the ordering of distributed events in time—synthesizing concepts from discrete-event co-simulation and dataflow programming for distributed systems [29, 30, 31]. These concepts will be introduced incrementally and in greater detail throughout this section.

### 2.1 Process interface

Processes are the operational units of integrative models, each simulating distinct mechanisms that drive changes to the system. Considering the multitude of processes present in biological systems, it is beneficial to adopt a *process-centric* design when building models. By emphasizing the **process interface**, we enable modelers to scrutinize individual processes independently prior to their systematic integration.

A process interface defines the types of state variables that are exposed by the process—its inputs and outputs—and configuration settings. An interface definition can serve as a process type, where processes conforming to the same interface can be substituted reciprocally within a composite system—for example, different simulation methods that have the same interface and perform the same function could be swapped out for each other—much like different enzymes that catalyze the same reaction. Such interfaces could also represent specific biological phenomena—such as a gene regulation process that incorporates transcription factors as inputs and gene expression levels as outputs would define those variables in its interface. Processes can simulate different scales, such as one operating at an atomistic scale and the other at a coarser molecular scale, and these could be swapped out based on level of detail required.

Figure 3 depicts a process as a rectangle with input and output ports along its boundary. Mathematically in equation 1, a process is defined by an update function that converts input data from input ports into output data at output ports. Each process is configured by a “config”, which could include any constant, dataset, model file, or static data type that informs the process’s update. The update function takes in the inputs, proposes changes to states, and sends these updates through the output ports. Both the ports and the config are defined by types, which determine the acceptable values for inputs and outputs. Update functions are ideally pure functions that keep all state variables external, facilitating composition with other processes. The process interface includes the config and ports along with their types, as well as the update function, which maps inputs to outputs, and can be formally depicted as follows:

(1) $$\text{process(confi}{\text{g}}^{\text{type}}):\left({\text{in}}_{1}^{\text{type}},{\text{in}}_{2}^{\text{type}}\right)\to \left({\text{out}}_{1}^{\text{type}},{\text{out}}_{2}^{\text{type}}\right)$$

By providing a general interface with standard methods of composition, we open the framework to all possible models—it could support a simulation module, configured by passing in parameters or model file, receiving initial conditions through the input ports and returning simulation results as an output. It could be a translator, which reads an input dataset and converts it to a format understood by a different simulator. It could be an cell agent, with a specific cellular phenotype specified in the config, and an update function that maps its sensory or chemical inputs to behavioral outputs. It could be a neural network, configured by weights and other parameters. Or a figure generator that reads the simulation state as input, and returns a rendered figure as output. The options are endless, keeping the framework open-ended.

**Stores** facilitate interactions between processes by holding and managing the externalized variables, and serve as conduits through which processes connect. Graphically represented as circles in Figure 4, stores are nodes that hold values defined by data types. In a type system, each type dictates how processes can interconnect; processes can only link to a store through a port that share the same type, ensuring coherence. Types may capture information such as units (meters, moles, liters), data types (integers, arrays, dictionaries), or more complex types like position coordinates (x, y, z) or images. By reporting the type of our datasets, we can implement accurate connections and facilitate automatic composition. Stores can also be nested within each other using a place graph (Fig. 4BC)—sometimes called a tree or forest graph—this format facilitates the structuring complex biological data into a format that reflects the hierarchical organization of biological states.

### 2.2 Composition patterns

Composition patterns, or “wiring”, details how process connect by their ports to shared stores, establishing relationships between their interfaces. When connecting stores and processes at the same level, without nesting, wiring determines a pure **process graph** in which multiple processes can connect to stores through their ports with matching types (Fig. 5A). When there is a hierarchy, processes can connect across levels, and can translate states at the micro-level to the macro-level, or set top-down constraints—the composition of a place graph and process graph is a process bigraph (as shown in Fig. 2B). Distinct processes that declare the same interface can be interchanged to fulfill these relationships, thus allowing the simulation engine to swap out processes with comparable interfaces.

A composite, made of multiple processes, can itself be a process (Fig. 5B). This means a composite has its own interface and can be embedded in a higher-order composite. To achieve this requires adding inwardfacing ports that connect to the internal stores—as shown by the red wires in Fig. 5B—which synchronize to their corresponding external store, connected through external ports across the interface. A composite needs to implement an orchestration pattern that determines the order by which it triggers internal processes (discussed in 2.3). The possibility of composite processes means we can spin up entire hybrid simulations and plug them together as modules of a super-simulation — an integrative multiscale simulation *within* an integrative multiscale simulation that can run on a separate computer and only synchronize the required states across the composite interface. This is why process-bigraph is not a black box approach—it can replace simple processes with composite processes that have matching interfaces—equivalent to zooming into composite processes to reveal their internal structure, function, and orchestration.

### 2.3 Orchestration patterns

Composite simulations host numerous interacting processes that operate concurrently and across timescales, each fulfilling unique functions. These processes are managed by the composite’s update function—its simulation engine—through **orchestration patterns**, which determine their ordering, triggering, and result-gathering. Composites act as orchestrators, following these patterns to select processes, project the simulation state to their ports, trigger their updates, collect and project the updates back, and apply them to the internal simulation state.

There are several useful orchestration patterns included in Vivarium, all illustrated in Figure 6. **Multi-timestepping** (Fig. 6A): asynchronous processes update at their preferred timescales. They are managed by a discrete-event co-simulation method that schedules the time-to-next update, collects the results, and triggers each process to run in parallel. **Workflows** (Fig. 6B) use a directed acyclic graph to set the order of process updates, with each triggered by the completion of preceding processes, or by changes to upstream stores. **Event-driven graph rewrites** (Fig. 6C), sometimes called reactions, are updates that restructure the composite’s graph—this can include moving stores or processes, adding and removing stores or processes, or rewiring process ports to different stores. Different orchestration patterns can be composed together and made interoperable, for example having workflows and graph rewrites running between temporal processes, or entire integrative simulations running as a step of a workflow. The exact sequence and manner in which they execute should emerge dynamically from the properties of the individual processes (such as preferred time scale or update conditions), and their composition pattern determines which states they respond to.

Additional methods of orchestration can include the management of ensemble models, with many parallel simulation of model variants running in parallel, and a step process that takes the results and integrates them into a single unified prediction. Biomedical digital twins [32] require ongoing streaming input, and require orchestration that can continually update the composite’s parameters, processes, and overall structure to match observations as they become available. Discovery engine composites may run inference on their internal models, and report new parameter values or other inferred knowledge through their output ports.

### 2.4 Standardized composition protocol

A composition protocol defined by standardized schemas for process interfaces, composition patterns, and orchestration patterns, can form the foundation of robust infrastructure for compositional systems biology—a project currently underway that will be described with the release of the process-bigraph software suite. Schemas provide structured data formats that that can ensure reproducibility, compatibility with different tools, and enable “plug-and-play” integration of new processes and data into ongoing simulations. Different software tools built around a common set schemas can include databases, applications, user interfaces, and tools; they can be supported by local and remote operations, including containerized and web-based services.

A standardized composition protocol could reduce the fragmentation that hinders collaborative advancements. By aligning with existing standard formats like SBML or CellML for biological network models [33, 34], standard formats for spatial models [35] and multi-cellular models [36], the BioModels database for existing models [37], the BioSimulators database for simulation tools [27], and SimService for containerized simulation modules [38], the protocol can foster a unified approach that lets these efforts connect with others. There is much work to do—each tool would need report its interface, and datasets would need to report matching type information so they can be accurately connected to. A unified framework for composition will accelerate innovation and drive significant breakthroughs in systems biology research, addressing many challenges by advancing the FAIR (Findable, Accessible, Interoperable, Reusable) [39] principles, which will allow researchers to more reliable find simulation modules, understand those models, and connect them reliably into hybrid, multiscale models.

## 3 Templates for multiscale cellular modeling

With the conceptual and graphical framework for composition in place, we can begin the compositional analysis of cells—a similar approach could be taken for different biological scales, such as tissues, multicellular organisms, or whole ecosystems. A compositional analysis of cells requires taking a dual perspective, of the cell as a process interfacing an environment, and of the cell as a composition of interacting molecules. By taking these perspectives simultaneously, we ask: How does the cellular interface emerge from interactions in the molecular domain? How is this interface maintained and remodeled by processes internal to the cell? How does this interface respond to environmental changes? What happens to this interface upon cell death? Growth and division introduce additional nuance, driving multi-cellular compositions to build up into heterogeneous multi-layered structures that undergo development and evolution.

While this section does not present quantitative models, it offers high-level templates for such models—schemas of subsystems and the connection between them, which can later be populated with data and mechanistic details and should be advanced by an open community of researchers. Templates aim to guide the construction of more detailed models by first defining their input/output relations and translations, and only afterwards adding the detailed mechanisms and fitting them to experimental observations. As a bonus, examining these templates from a compositional perspective, without delving into implementation details, provides valuable insights into how these systems interconnect, function, and evolve.

### 3.1 Cellular interface

Differentiating a cell from its environment is easy to do observationally—every cell has a membrane that separates it from its environment. The membrane serves as an interface, through which the cell and its environment interact, involving the transport of molecules through channels, the binding of signals to membranebound proteins, the maintenance of a voltage gradient across the membrane, the application of cytoskeletal forces that stretch and shape the membrane, responding to forces and fluxes exerted by the environment. Characterizing the cellular interface requires us to be explicit about its inputs and outputs (Fig. 7); this includes listing all variables that couple the internal state of a cell to its environment and including their type information, such as units and other constraints.

There have been several initial efforts to characterize cell behavior for the purpose of multi-cellular modeling. This includes the Cell Behavior Ontology [40] and MultiCellDS [41], which includes object descriptions for interface qualities such as cell state, border, membrane parts including adhesion, gap junction, channels, receptors, and pumps. However, these same efforts describe internal cellular and environmental components, as well as multicellular interactions without clearly distinguishing interface elements or providing robust type definition for the data associated with these components. Compositional systems biology should adopt as much of these original categories so that established knowledge is preserved, but there will need to be an effort to sort them into composable types.

Initial efforts to characterize the cellular interface should focus on identifying a core set of elements that are ubiquitous across cell types. We can consider the most basic and universal interface shared by all cells: a minimal cell interface (Fig. 7A,C), serving as the foundation for more differentiated cell models. This minimal cellular interface can also represent a common ancestral form of cells, which included the minimal necessary interfaces that allowed early cells to survive—this likely includes minimal forms of membrane and metabolism with consumption and secretion for basic maintenance. This requires ports for chemical fluxes, which includes uptake of nutrients or exudation, a mass of the cell, which is felt by the environment, a cell shape which may default to a sphere, and viability bounds (sometimes called “essential variables” [42]) which define the range of environmental conditions within which the cell can survive.

A more differentiated cellular interface (Fig. 7B,D) refines the minimal interface, sub-categorizing the different ports and may add new ports entirely—such as light sensitivity, genetic exchange, and electrical conductance This includes specialized molecules for surface forces, such as adhesion molecules (e.g., cadherins, integrins), motility-generating molecules (e.g., myosins, kinesins, dyneins, flagella), mechanical tension elements (e.g., cytoskeletal elements, extracellular matrix interactions). Flux ports become more sophisticated, subdividing the port according to nutrient transporters (e.g., glucose transporters), waste removal mechanisms (e.g., exocytosis, efflux pumps), and signal transduction (e.g., G-protein-coupled receptors, receptor tyrosine kinases). By meticulously defining each of these interface port types, we would transform the cellular interface into a robust, composable model that lays the groundwork for future refinements that will help us decipher the evolutionary expansion of cellular interfaces.

The cellular interface not only sets the inputs and outputs of a cell but also imposes constraints on the update function mediating these transactions. Essential among these are mass balances, which ensure that the cell neither creates nor destroys mass—each unit of mass acquired from the environment, an input, directly contributes to the cell’s mass, an output as seen by the environment. The interface regulates various types of fluxes: chemical exchanges, mechanical forces, electrical currents, and thermal energy transfers (potentially informed by bond-graphs [43]). Viability bounds set constraints on allowable environment conditions—if these bounds are crossed, the cell dies and ceases to have an interface.

### 3.2 Connecting to an environment

Contact with an environment dictates which specific fluxes the cellular interface is exposed to, driving its dynamics and behavior. Setting aside coupling with external cells for later discussion, we consider a single cell in an abiotic environment that may include chemical, electrical, mechanical, light, and thermal gradients, as well as physical barriers (Fig. 8). Often in a simulation, environmental states are represented as a field with concentrations of molecules, or discrete objects that can create mechanical obstacles for the cell; in non-spatial simulations they may just be a pool of available resources. Environmental processes may include diffusion and advection of the fields, and mechanical processes that exert physical forces.

Connecting to environmental states create potentials across the cell interface, driving inputs that orchestrate internal cellular processes. The environmental state and the processes driving those states, can cause alterations to a cell’s gene expression, metabolism, and cell shape. Conversely, the cell actively shapes its surroundings by secreting extracellular matrix components, exerting mechanical forces, adhesion to external surfaces, and modifying structural elements by releasing enzymes such as proteases and hydrolases, and redistributing nutrients and signaling molecules. This creates feedbacks that are sensorimotor and also chemical. This bidirectional interaction is critical for the cell’s ability to adapt to changes and construct its niche, seeking out desired states as seen through its interface.

At this stage, composite models of cells and environments may resemble ABMs, which emphasize a cell’s behavioral responses to its surroundings, simplifying or ignoring the underlying molecular processes. For instance, an ABM might establish rules for a cellular update function, such as moving towards or consuming nutrients based on the cell’s internal state of hunger and environmental cues. Decision-based models enhance this by incorporating a logic that prioritizes actions based on their utility—a configuration that maps a cell’s input states to some value or reward that needs to be maximized, such as growth, robustness, or replication. However, being more phenomenological, these models fall short in detailing the molecular mechanisms that drive a cell’s behavior, sensorimotor functions, or decision-making processes, and they do not explain the fundamental reasons why a cell *needs* nutrients in the first place. To delve deeper into these aspects, we need to shift to a molecular perspective.

### 3.3 Molecular interface

When we peel away the membrane, we see the cell is itself a composite system of many interacting molecules, which can be associated in larger assemblies such as complexes, condensates, and organelles. Molecules interact with each other through various mechanisms, including chemical reactions, binding, transport, electrostatic interactions, mechanical forces, and so forth. To understand how systems of molecules compose to create a cell, we first need to characterize the molecular interface.

A molecule can engage with other molecules in many ways, but contemporary models often focus narrowly on a subset of this interface. For instance, molecular models may focus on the kinetics of a reaction catalyzed by a specific protein, while ignoring its structural properties or spatial location. Reducing the interface simplifies a model and enhances computational efficiency but also limits its scope and ability to compose in the same way real molecules do since they can just use the available physics while models require explicit definition. Composition can overcome this by integrating elements from different modeling frameworks to create a more comprehensive representation of the molecular interfaces. There has already been substantial progress in integrative modeling for the structure of molecules [44]; we also need to include functional models. Composition could bring together structural models with molecular dynamics, kinetic reaction networks, particle-based stochastic models, coarse-grained models, continuum models, etc. Each of these frameworks provides unique insights and can be composed to reflect the complex interplay of molecular interactions within the cellular environment.

Ports can be further subdivided according to the molecular mechanisms. For example, enzymatic reactions (Fig. 9C) are a differentiated, specialized cell mechanism, which divide chemicals/molecules into subtypes: 1) substrates: molecules that enzymes act upon, 2) catalysts: enzymes or other molecules that speed up reactions, 3) cofactors: non-protein molecules that assist enzymes, 4) products: molecules produced from enzymatic reactions.

A comprehensive molecular interface definition would encapsulate all the types of interactions possible between molecules, representing them as processes with defined ports (Fig. 9). Each process supports various interaction types, including chemical reactions that form or break chemical bonds, leading to the creation of new molecules. Physical interaction processes, such as van der Waals forces, hydrogen bonding, and ionic interactions, are also integral. Electrostatic interaction processes are characterized by attractive or repulsive forces between charged ions. Hydrophobic interaction processes play a crucial role in protein folding and stability. Steric processes affect molecular size and shape, augmenting their reactivity and stability. Additional input ports can include conditions that influence molecular interactions, such as temperature, pH, and mechanical forces, allowing for dynamic modulation of these processes.

### 3.4 Biomolecular compositions

We can begin assembling molecular processes through their shared states, creating larger and more intricate assemblies (Fig. 10). There are many ways to approach this task—it can include the progressive composition of metabolic pathways [45], or lipids [46], it could take integrating the spatial distribution of proteins with their interactions [47], or taking individual molecular structures and packing them together into a volume [48]. Each molecular process is defined by specific ports—such as active sites on enzymes, binding sites on receptors, and docking domains on structural proteins—which facilitate the coalescence of molecules into functional complexes. These interactions are orchestrated through a set of molecular affinities and repulsions, aligning and connecting molecules to achieve functional activity. As these complexes integrate into even larger compositions, they can form condensates through mechanisms like liquid phase separation [49]. Spatial segregation by membranes encapsulates these assemblies into organelles, creating subcompartments with distinct internal molecular compositions and boundary conditions.

In the task of assembling molecules, as we approach the scale of a whole cell, the challenge of recreating the cellular interface from molecular components becomes evident, as the boundary between internal and external states remains ambiguous. For Figure 10 this would take drawing a process rectangle around a subset of the shown states that can specify a minimal cellular interface, which requires delineating the molecules and processes within the cell from those outside of the cell. Each molecular mechanism—whether transmembrane transport, gene expression, protein synthesis, signal transduction, metabolism, protein folding, or DNA replication—lacks a clear distinction between whether it is inside or outside a cell. Processes such as transport reactions, membrane trafficking, and the production of extracellular matrix can seamlessly link across what we conventionally consider the cell boundary. Despite our ability to assemble molecular components into large-scale composite structures, systems biology still struggles to define what makes one particular molecular composition a living cell and the other one dead matter. It is something about the specific arrangement of molecular mechanisms allows them to orchestrate activities in a way that is consistent with that of a living system, according to a cellular interface. Without a clear framework to distinguish living molecular compositions from non-living compositions, we cannot objectively determine which individual we are observing or how it behaves within its environment is oriented to meet its needs.

### 3.5 Self-organization and the emergence of the cellular interface

One challenge in multi-scale modeling lies in determining when to coarse-grain complex processes and when to revert to detailed molecular parts (Fig. 11). Coarse-grained processes allow for more intuitive modeling at a scale that matches experimental observations, can integrate macroscopic variables, and is computationally less-expensive. This decision is crucial for capturing the dynamics of self-organized biomolecular systems, which rise from independent molecular processes, but through their composition determine an organization that can be abstracted from its molecular grounding.

Self-organized molecular processes form when composites of molecular processes harness external energy gradients, and use these to maintain order far from equilibrium. In this state, energy flows create work energy that maintains the system’s organization [50], much like a hydroelectric dam or a fluid vortex. When such processes form, we may want to identify them as emergent self-organized processes that have their own properties, and abstract away the underlying molecular processes from which they emerge [51]. A selforganized process would have an entropy port, to release into the environment, and a work energy port, which feeds off the external gradient. However, as the external gradient depletes, a molecule system decays back to equilibrium and the self-organized process ceases to exist, and is replaced again by independent molecular processes.

Autopoiesis is a unique form of composition where two or more self-organized processes are constrained by each other in a way that keeps their gradients active, enabling continuous self-construction. Processes in an autopoietic composition form a network of self-organized molecular processes that continually re-synthesize their required materials and maintains a boundary separating themselves from the environment [52]. The mutual support of metabolism (Fig. 11A, top) and containment (Fig. 11A, middle) exemplifies how the output of one process creates the constraints required by another: autocatalytic networks counter molecular degradation through catalysis, while self-assembled lipid membranes provide spatial closure to counteract diffusion. Closed membranes allow for proton-motive force and other chemical gradients to be maintained by the system. Replication (Fig. 11A, bottom) is a process by which a template of DNA or RNA is copied. Their mutual support enables molecular systems to persist and self-organize as distinguishable entities.

This again raises the question of a minimal cellular interface—what are the minimal sets of self-organized molecular processes that can together recreate a cellular interface? A minimal autopoietic composition can also represent a common ancestral form of cells, which included the minimal necessary interfaces that allowed early cells to survive—this likely included minimal forms of containment, metabolism, and replication [53].

Coarse-graining presents the challenge of maintaining the integrity of cellular models while recognizing the necessity of decomposition back to molecular parts due to the fundamental nature of cell disintegration. While stable emergent cells can be represented with fixed organization in coarse-grained models, this approach risks overlooking the precariousness of cellular life [54]. By coarse graining over molecular processes, we may be inadvertently removing the capacity for disintegration from the cell model, obfuscating the processes separating living and nonliving organizations A cell must maintain homeostasis, if its balance of required processes are ever broken this leads to irreversible disintegration driven by entropic forces. To accurately capture cellular dynamics, models must incorporate the ability to switch between detailed molecular representations and coarse-grained abstractions, reflecting the critical processes of both self-organization and disintegration.

### 3.6 Growth, division, development, evolution

As cells absorb nutrients and convert them to biomass, they increase in mass, volume, and surface area. This can drive changes in variables such as surface area to volume ratio, which influence how effectively cells can interact with their surroundings; a lower ratio can reduce the efficiency of surface-mediated cellular responses. To circumvent this challenge, rod-shaped bacteria maintain a relatively stable surface-to-volume ratio by elongating, with a fixed width (Fig. 12A,B). Growth also leads to compositional transformation within the cell—new proteins, lipids, metabolites, and other molecles are synthesized, diluting the concentrations of prior synthesized molecules, and altering the cell’s overall composition and functional capabilities [55, 56, 57].

Following cellular growth, division often ensues, marking a topological transformation where one cell interface bifurcates into two. During division, both the membrane and cytoplasm undergo reorganization, each molecule either goes into one daughter or the other. This creates two unique compositions, two distinct daughter cells, each equipped with its own interface. These new interfaces enable the daughter cells to connect independently with their environments, yet they often remain coupled due to spatial proximity, influencing each other as integral components of a shared environment. Cell division sets the stage for the formation of larger, heterogeneous cellular assemblies, possessing diverse interfaces that interact with varying environmental pressures.

There are different ways cellular interfaces can interconnect with each other through processes of adhesion, predation, contact, communication; these can range from simple interactions between two bacterial cells to complex ecosystems with many cells, including organisms made of many specialized cells. Symbioses include mutualistic, commensalistic, or parasitic interactions, forming stable compositions of cellular populations. As an example in bacteria, the development of multicellular structures seen in biofilms, where communal life is distinct from that of free-living bacteria [58, 59, 60], demonstrates how a multicellular composite exhibits emergent properties such as cooperation, resource capture, and increased survival under stress. The compositions produce a physical scaffold of extracellular matrix, holding them together and attaching them to environmental surfaces, and driving physiological changes to their constituent populations.

As cellular populations expand through growth and division, the heterogeneity within them enhances their capacity to adapt (Fig. 12C,D). Fitter interfaces, which exhibit enhance growth and division by effectively connecting to their environments, are naturally selected for their efficiency in resource utilization, environmental resilience, and intercellular communication. Cells that fail to meet their viability constraints are pruned out of the composition as their processes disintegrate and their contents are redistributed. An developing multicellular composition integrates new processes, continually restructuring itself to optimize energy flows and resource management across the entire system. The incorporation of new modules introduces novel capabilities and redefines pathways of energy and resource distribution, augmenting the functional complexity of cells and shifting the overall dynamics within the population. Evolutionary pressures meticulously refine these compositions, fostering the development of more efficient and resilient cellular systems. A dynamic multicellular composition, driven by environmental interactions, constructs sophisticated cellular structures that are optimized for energy flow and functionality, thereby enhancing the survival and adaptability of the organism and forming the basis for evolutionary advancement in diverse environments.

## 4 Collaborative biosciences

The composition framework and biological templates introduced in this essay offer the foundation for an ambitious project to model multiscale cellular systems with increasing molecular and physical detail through iterative, community-driven refinement. For this final section we focus on scientific collectives—teams of scientists, experimental and computational technologies, the biological systems under study, and the resulting models that are created through our coordinated efforts. In many ways, these scientific collectives can also be viewed as compositions that that are orchestrated by the activities of many human cognitive processes, which connect to each other and to our shared models via different available interfaces whether it is scientific papers, software APIs, or visualizations (Fig. 13). We should ask: how can we optimize these compositions to efficiently build more realistic and accessible models?

Enhancing the productivity of individual human contributors is critical. We need to access data more easily, update our models more efficiently, test these models, and set off experiments to validate and to collect more data. Improvements could be made with human-centered design, studying the human interface and how it connects to the models and to the real biology in question. We should be developing intuitive software and visualization tools that align with scientists’ workflows—such as Jupyter notebooks, GUIs, or chatbot assistants—enabling easier navigation and control over complex datasets and models. Gamification could be leveraged to enhance engagement and problem-solving for scientific discovery [61]. Human-in-the-loop approaches ensure these tools support real-time interaction and feedback, integrating seamlessly with ongoing model building and experimental design.

At a more organizational scale, our collaboration strategy should allow each scientist to layer their data and models seamlessly and ask advanced questions of a biological simulation. The integration of tools such as GitHub facilitates dynamic collaboration and evolution, making the ecosystem more accessible and intuitive. An open composition standard ensures seamless interaction between various tools and systems, allowing for custom interfaces and improved modes of interaction.

Scientific collectives, such as consortia, catalyze transformative advances by enabling the integration and nuanced analysis of vast, heterogeneous datasets. These consortia foster a culture of team learning and collective problem-solving, pooling resources, data, and expertise to tackle complex challenges. By organizing symposia and workshops, consortia facilitate crucial exchanges that drive the project forward and embody the compositional nature of our scientific approach. A specialized consortium using a compositional framework reflects a cellular system, combining expertise from different levels with personal interfaces to build the integrative multiscale model, leveraging collaborative dynamics to address the multi-layered challenges of modern biology.

By continuously integrating new models and data with traditional methods and established knowledge, we advance towards a comprehensive model of life, driving scientific progress through structured, collaborative efforts that layer and rigorously test knowledge, all facilitated by the principle of compositionality.

## Figures and Tables

**Figure 1:**
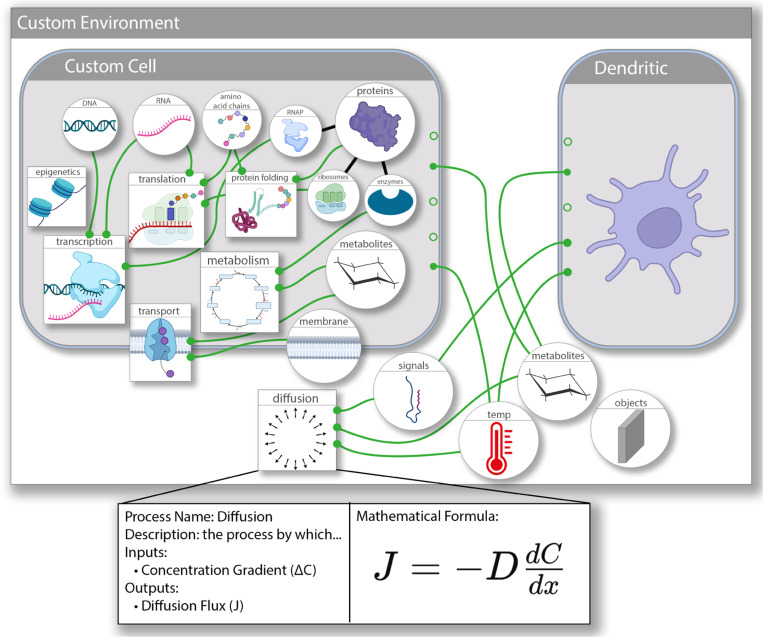
Conceptual design of a user interface for compositional systems biology. This illustrates how cellular systems can be modeled through the composition of submodels connected by wires. The graphics display nested modules, with multiple cells within an environment, and internal to the custom cell processes like metabolism, transcription, translation and protein folding. The zoomed-in diffusion process displays model information. Additional interactive elements may include clickable user elements to run the simulation, menus, and drag-and-drop interaction for intuitive model construction. Making this vision a reality requires a robust compositional framework.

**Figure 2:**
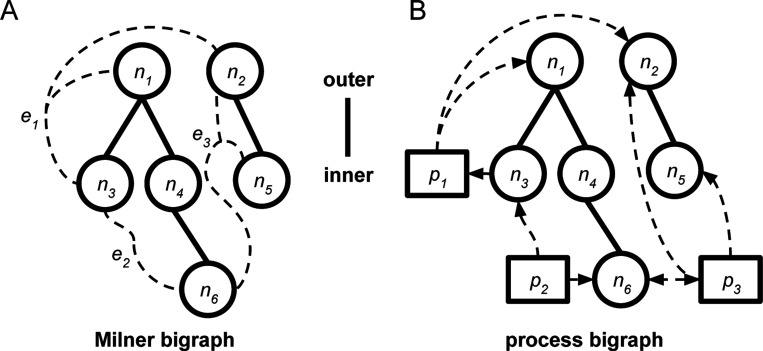
The original and extended bigraph structures. a) Milner bigraphs are made of a link graph with hyperedges (*e*_1_, *e*_2_, *e*_3_) and nodes (*n*_1_, *n*_2_, *n*_3_, *n*_4_, *n*_5_, *n*_6_), and a place graph connecting the nodes—place graph edges (unlabeled) are shown as thick black lines delineating nesting relation, with outer nodes shown above and inners nodes below. b) A process bigraph replaces the link graph with a process graph, made of processes (*p*_1_, *p*_2_, *p*_3_) connecting to the nodes through their ports—the direction of these ports shown by the arrow direction of each wire coming from each process.

**Figure 3:**
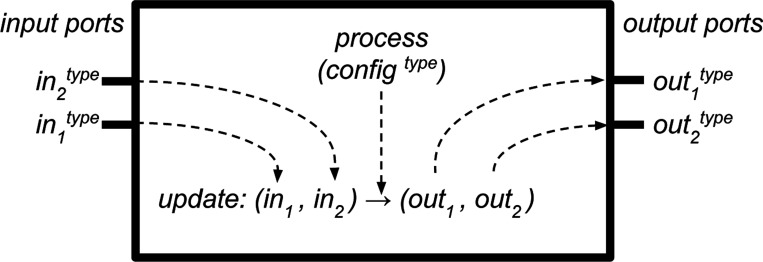
Process diagram. A process is depicted as a rectangle, with ports along its boundary to represent its interface. Here, input ports are shown on the left and output ports on the right—this convenient visualization style is not enforced, and for simplicity one may choose to place the ports anywhere on the boundary, possibly using arrows to signify whether they are inputs or outputs. If no arrow is shown on the port, it is assumed to be bi-directional. Ports also specify what type of information can flow in and out—these types are shown in superscript, and are in this figure unspecified as a generic type called “type”. The update function is a mapping from inputs and outputs, informed by the config.

**Figure 4:**
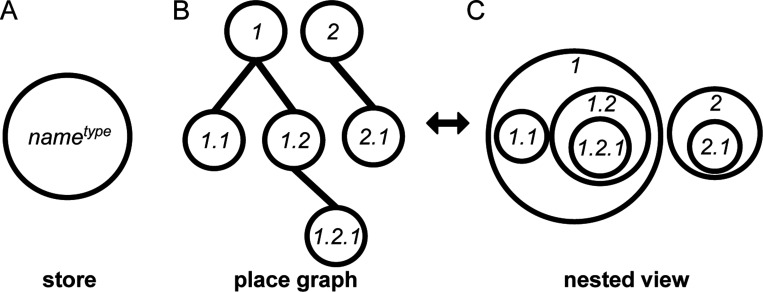
Store diagram. Stores, shown as circles, can hold any data type and get nested in hierarchies to support multiscale representation. A) A basic store, showing the store’s name (“name”) and the data type it holds in the superscript (here an unspecified “type”). B) Nested stores in a place graph, with outer stores above, connected by a thick black edge to their inner stores below. C) The same place graph from B is depicted in a nested view, with inner stores placed within their outers.

**Figure 5:**
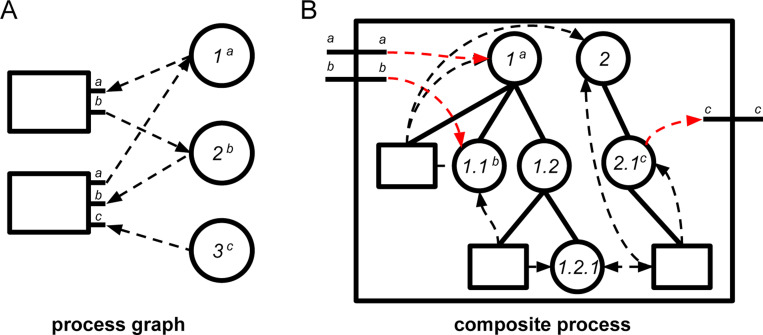
Composition patterns. A) A process graph is made of processes connecting to stores through their ports. All ports for the processes have a type that must match the store type it connects to (shown with the types *a, b, c* on the ports and the stores), and inputs/outputs are here shown by the direction of the arrow on the wire coming from each port. These stores are not nested, so no place graph is required—this composite reduces to a process graph. B) A composite process, which has a process bigraph running within it and which it orchestrates, and has its own input and output ports that can connect to external stores. For every external port, a composite process also has internal ports with matching types that connect to the internal composite, as shown in red.

**Figure 6:**
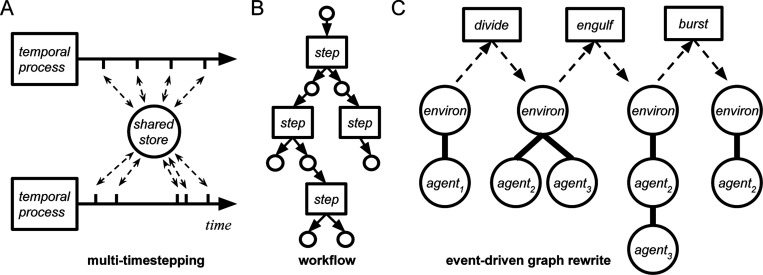
Orchestration patterns. A) Multi-timestepping, with temporal processes each updating at their preferred time intervals, orchestrated by a discrete-event co-simulation engine. B) A workflow is a directed acyclic graph that sets the order of updates for step processes, with each one triggered by changes to its input states. C) Event-driven graph re-writes orchestrated as instantaneous events, change the topological structure of an agent-environment system. Each graph re-write can be specified as a reaction, triggered by states internal or external to the agents in the environment. “Divide” makes one agent divide into two. “Engulf” places one agent within the other. “Burst” dissolves the agent, releasing its inner components back to the environment.

**Figure 7:**
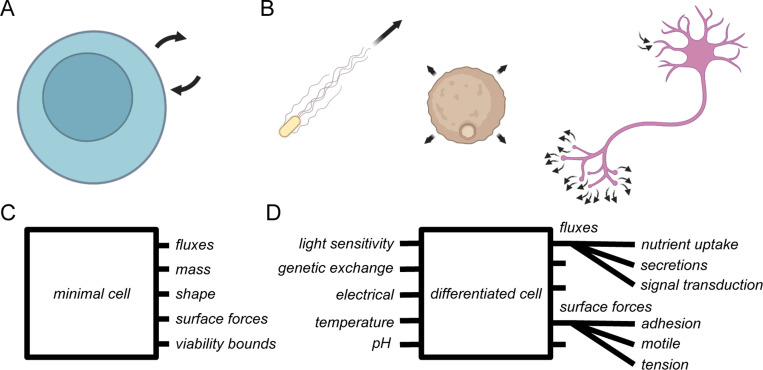
A cellular interface defines the input/output relations of a cell. A) A minimal cellular interface represents the minimal required properties of cell model, which all cells must uphold. B) Examples of more differentiated cells with their own unique interfaces—*E. coli*, yeast, and a neuron. C) A minimal cell interface diagram, showing some required ports—fluxes, surface forces, and temperature. D) A more developed cellular interface includes additional ports and refinements of existing ports. The fluxes port is subdivided into nutrient uptake, secretions, and signal transduction. The surface forces port includes adhesion molecules and mechanical tension. New ports include light sensitivity, genetic exchange, and electrical conduction.

**Figure 8:**
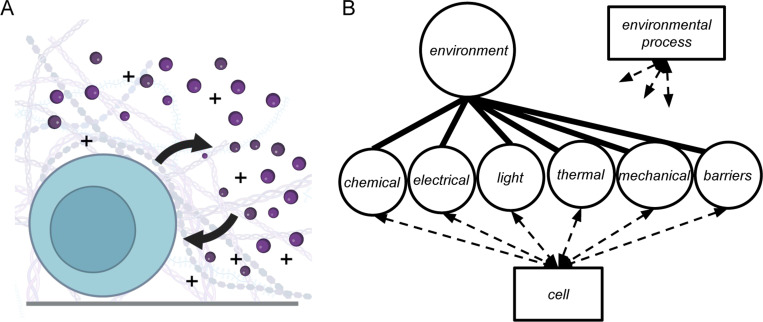
Cell-environment composition. A) Illustration of the cell connecting with external states such as molecules, electric charge, an extracellular matrix, and obstacles such as a floor. These environmental states may be shared with environment processes. B) Composition diagram, showing the cell interacting with its environment through sub-states involving chemical, electrical, light, thermal, mechanical interactions, and barriers.

**Figure 9:**
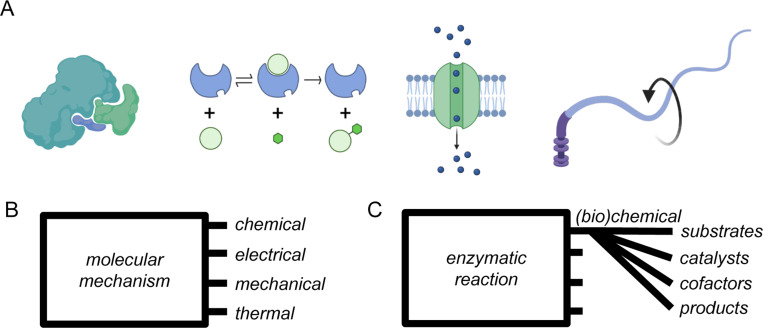
Molecular interfaces are processes that have molecules as inputs and operate by changing the molecular states. A) These can take a range of forms: a structural model of how molecules fit together, enzymatic reaction with an enzyme attaching two small molecules, a transmembrane transport process, or motility-generation with a flagella’s production of thrust. B) A molecular interface diagram showing basic molecular properties with their own types and constraints: chemical, electrical, mechanical and thermal ports. C) A more specialized molecular mechanism could subdivide any of the ports, such as the enzymatic reaction differentiating between the substrates and cofactors that are taken as input into the reaction, a catalyst, and the resulting products and changes to the substrates as outputs.

**Figure 10:**
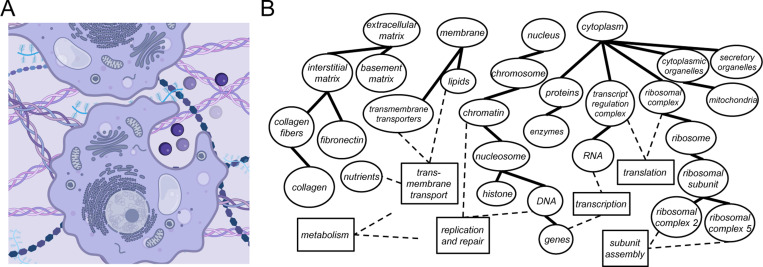
Molecular compositions as a nested composites. This figure illustrates the hierarchical organization of molecular components that exist both within and outside of cells. A) Different molecular structures both within and external to a cell, including a membrane, organelles, nucleus, and extracellular matrix. Complexes such as the ribosome complex can be subdivided into smaller complexes, all the way down to individual proteins. B) Composite processes diagram showing nested structure of a cell, with circles representing nested states and rectangles representing processes. Each process, such as transmembrane transport, transcription regulation, and protein folding, connects with specific nested states like the membrane, cytoplasm, and nucleus. These interactions facilitate various cellular functions, from maintaining molecular gradients to coordinating complex biological activities within distinct subcompartments.

**Figure 11:**
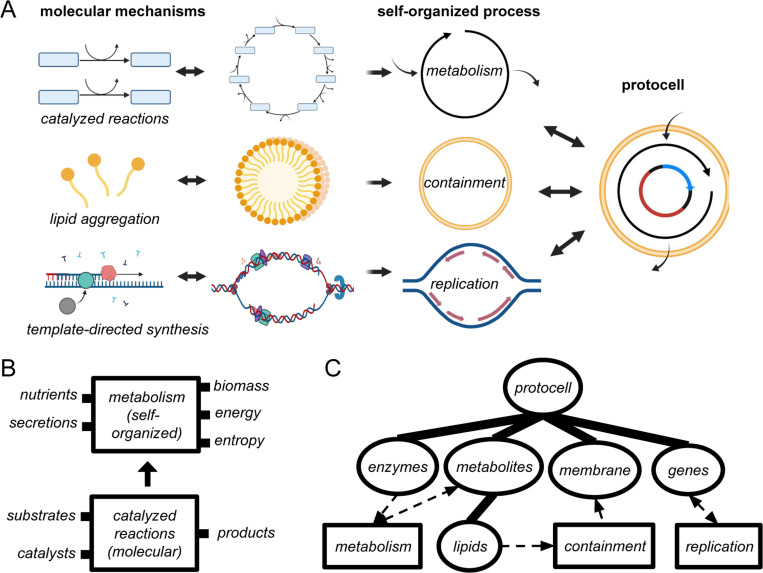
Self-organization and autopoiesis. A) Several self-organized processes: autocatalysis, membranes, and template replication compose into a self-sustaining autopoietic organization. B) Diagram showing the transition from spontaneous catalyzed reactions—a molecular process—to a self-organized metabolism process made of several catalyzed reactions that form a close loop. C) A protocell composition diagram with minimal required metabolism, containment, and replication processes, supporting each other through catalysis of required parts, containment to counter diffusion, and replication to support the generation of new protocells.

**Figure 12:**
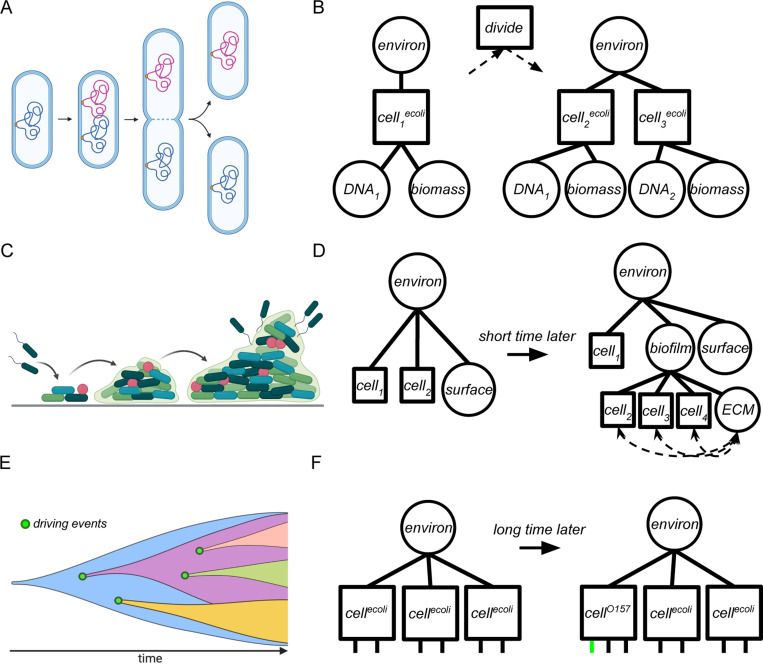
Growth and division leading to evolution. A) Prokaryotic binary fission: cell initiates replication copy of DNA is created cell elongates, cross wall forms daughter cells separate. B) These are hierarchical updates, with one cell before that has some DNA and biomass, resulting in two cells afterwards with a new copy of the DNA and each with its own biomass. C) Biofilm development, initiated by free-living bacteria that attach to a surface, secrete ECM, and grow with functional specialization. D) The development of biofilm includes a hierarchical change, in which free-living bacteria come under a unified biofilm composition in which multiple cells are bound together by an extracellular matrix (ECM). E) Evolution over time, with key driving events shown in green, leading to new functionality that expands in the population over time. F) The driving event may come in the form of a new interface port added to a cell, shown in green. This can give the cell new ability to sense or influence the environment, leading to evolutionary expansion.

**Figure 13:**
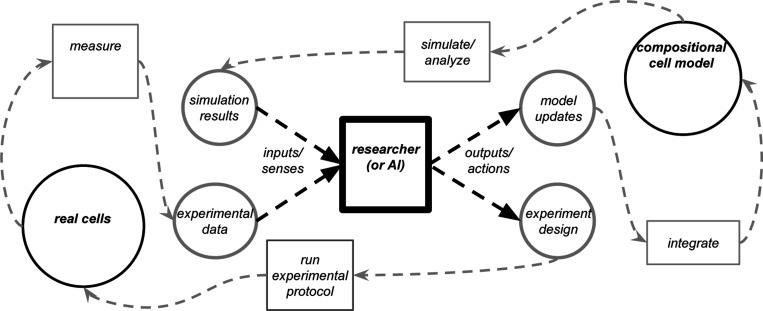
The extended interface of a human researcher, which connects real cells with models. Our inputs come in through our senses—sight, audition, touch—which in the context of modeling typically comes through our digital devices—a screen or speaker. Our outputs are actions—speaking, typing, clicking with a computer mouse. These interactions connect us to real cells via experimental technology, and to our models via our computers, as well as the extended internet as a knowledge base. It is our job to build the best models to match our experimental observations, and to optimize the production of models requires a clear strategy for how we best compose with this distributed infrastructure.
